# Irrelevant stimulus processing in ADHD: catecholamine dynamics and attentional networks

**DOI:** 10.3389/fpsyg.2014.00183

**Published:** 2014-03-26

**Authors:** Francisco Aboitiz, Tomás Ossandón, Francisco Zamorano, Bárbara Palma, Ximena Carrasco

**Affiliations:** ^1^Department of Psychiatry, Medical School, Centro Interdisciplinario de Neurociencia, Pontificia Universidad Católica de ChileSantiago, Chile; ^2^División de Neurociencia, Centro de Investigación en Complejidad Social, Facultad de Gobierno, Universidad del DesarrolloSantiago, Chile; ^3^Programa de Doctorado en Psicoterapia, Pontificia Universidad Católica de ChileSantiago, Chile; ^4^Servicio de Neurología y Psiquiatría, Hospital de Niños Dr. Luis Calvo Mackenna, Facultad de Medicina, Universidad de ChileSantiago, Chile

**Keywords:** attention, CNV, fMRI, P300, ventral attentional network

## Abstract

A cardinal symptom of attention deficit and hyperactivity disorder (ADHD) is a general distractibility where children and adults shift their attentional focus to stimuli that are irrelevant to the ongoing behavior. This has been attributed to a deficit in dopaminergic signaling in cortico-striatal networks that regulate goal-directed behavior. Furthermore, recent imaging evidence points to an impairment of large scale, antagonistic brain networks that normally contribute to attentional engagement and disengagement, such as the task-positive networks and the default mode network (DMN). Related networks are the ventral attentional network (VAN) involved in attentional shifting, and the salience network (SN) related to task expectancy. Here we discuss the tonic–phasic dynamics of catecholaminergic signaling in the brain, and attempt to provide a link between this and the activities of the large-scale cortical networks that regulate behavior. More specifically, we propose that a disbalance of tonic catecholamine levels during task performance produces an emphasis of phasic signaling and increased excitability of the VAN, yielding distractibility symptoms. Likewise, immaturity of the SN may relate to abnormal tonic signaling and an incapacity to build up a proper executive system during task performance. We discuss different lines of evidence including pharmacology, brain imaging and electrophysiology, that are consistent with our proposal. Finally, restoring the pharmacodynamics of catecholaminergic signaling seems crucial to alleviate ADHD symptoms; however, the possibility is open to explore cognitive rehabilitation strategies to top-down modulate network dynamics compensating the pharmacological deficits.

## INTRODUCTION

Attention deficit hyperactivity disorder (ADHD) is the most widespread childhood neuropsychiatric disorder, and is becoming increasingly recognized as a relatively common condition in adulthood (Aboitiz and Castellanos, 2011). A salient feature of ADHD is the inability to suppress irrelevant or distractive stimuli during a task. The current interpretation is that this and other symptoms are due to an impairment in behavioral and cognitive control mechanisms, caused by deficient dopaminergic signaling (Clark et al., 1987; Swanson et al., 2007). This pharmacological understanding has been complemented in the recent years with brain imaging and electrophysiology studies that have revealed deficits in the large-scale networks that regulate cognition and attentional control. However, we still have a large gap between neurotransmitter activity and network regulation in the cerebral cortex. The combination of both approaches may provide a multiple-level, unified interpretation of this condition, and in this paper we propose a tentative framework for this.

### DOPAMINE AND ADHD

Although it is widely accepted, there are many open questions concerning the dopaminergic hypothesis of ADHD. First, direct evidence for a specific dopaminergic deficit in ADHD is controversial (Krause et al., 2000; van Dyck et al., 2002; Jucaite et al., 2005; Spencer et al., 2005). Moreover, dopaminergic signaling is highly complex, involving different modes of transmission which are antagonistic between them and relate to different behavioral states (Seamans and Yang, 2004). On one hand, there is the transient release of dopamine produced by intense bursts of dopaminergic neural activity (phasic signaling). This modality is associated with salient sensory stimuli, and with attentional shifts and highly focused but short-lasting behavior. It has been proposed that an abnormal emphasis in phasic signaling is associated with impulsivity (Grace et al., 2007). Conversely, there is the basal or tonic signaling due to background levels of extracellular dopamine that change slowly over time. In the cerebral cortex, tonic or basal activity produces an overall decrease in the inhibitory tone of neural networks and has more complex behavioral manifestations. Abnormal variations in tonic signaling may have different long-term effects, including lack of motivation, distractibility or anxiety, depending of the level of basal activity (Aboitiz and Castellanos, 2011). Thus, ADHD symptomatology may not be simply explained by an overall dopaminergic deficit, but may rather reflect an altered signaling dynamics of this and other neurotransmitters.

### LARGE-SCALE BRAIN NETWORKS AND ADHD

Although the above approach provides pharmacological insight into the molecular and cellular bases of ADHD, at the same time it says little about the actual neural networks that become involved in the generation of specific symptoms. In the last years, a large number of imaging and neurophysiological reports assessing network dynamics in ADHD have been published, most of them focusing on brain activation during cognitive or executive tasks. These have revealed deficits in the activity of cortico-striatal systems that are key for sustained attention and goal-directed behavior (Swanson et al., 2007). However, a different line of research, triggered by the groundbreaking fMRI study of Raichle et al. (2001), disclosed a previously unknown network dynamics in the resting brain, called the default mode network (DMN). This network antagonizes with the “task-positive” networks that become activated during cognitive engagement. The latter include three main systems, the dorsal attentional network (DAN) involved in sustained attention and working memory, the ventral attentional network (VAN) involved in attentional shifts (Corbetta and Shulman, 2002; Corbetta et al., 2008), and the salience network (SN), responsive to emotionally relevant stimuli and related to pre-task expectation (Seeley et al., 2007).

The DMN has called widespread attention not only for providing a contrasting framework with task-positive networks, but also for its possible relevance in the generation of neuropsychiatric symptoms. For instance, a disbalance between the DMN and the task-positive brain networks has been considered to play a role in the distractibility of ADHD and other conditions (Sonuga-Barke and Castellanos, 2007). Normally, there is a strict antagonism between the default and the task-positive networks, such that activation of one is associated to strong inhibition of the second, a phenomenon termed anticorrelation. It has been postulated that in ADHD and in other conditions, the task-positive networks are unable to properly suppress the DMN during task performance, resulting in a coactivation of both networks and the rupture of the anticorrelation. Consequently, the stability of the focalized state becomes compromised. Distractibility might thus be a consequence of a bias toward the resting state condition and the incapacity to consolidate robust task-positive networks, due to interference with the DMN (Fassbender et al., 2009).

### HYPOTHESIS AND RATIONALE

**FIGURE 1:**
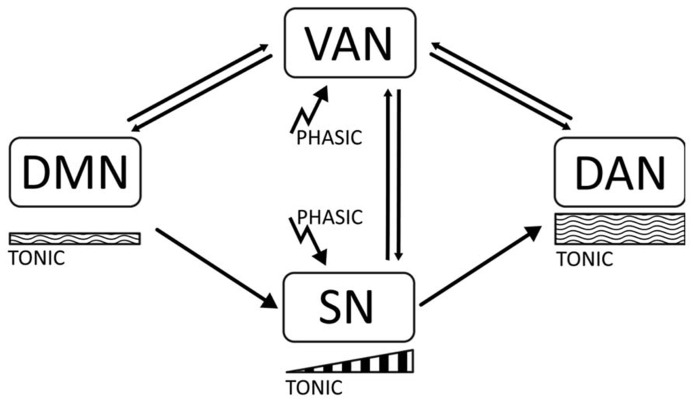
**The diagram represents the main proposals made in this article.** There are four cortical networks regulating behavior, the default mode network (DMN), active at rest; the dorsal attentional Network (DAN), active during task execution and involved in working memory and sustained attention; the ventral attentional network (VAN) involved in attentional shifting; and the salience network (SN) involved in task preparation. The DMN is associated with low tonic catecholaminergic activity, while the DAN is related to moderately high levels of tonic activity. Stimulus-related phasic catecholaminergic signaling activates the VAN, inducing attentional shifts in focused states and transitions between the DAN and the DMN. In addition, phasic signaling activates the SN, which is associated with a buildup of dopaminergic activity related to expectation and task preparation. In ADHD, insufficiently regulated tonic activity results in a disbalanced DMN and distractibility due to a low threshold for phasic signaling, which yields overactivation of the VAN during task performance, and short-term impulsivity. Dysregulation of tonic activity may also result in an attenuated SN due to an incapacity to build up mid-term tonic signaling for task preparation.

In this article, we will propose the hypothesis that there is a relation between the phasic-tonic balance of catecholaminergic signaling (dopaminergic and noradrenergic) and the dynamics between default and task-positive networks in the brain (Tomasi et al., 2009; Dang et al., 2012). Thus, stability of the task-positive networks DAN and SN may be associated to the maintenance of an appropriate arousal tone during the focused state, which is determined by moderately high tonic signaling. On the other hand, low tonic signaling may correlate with activation of the DMN. Phasic signaling may importantly serve to shift the attentional focus, and be related with the VAN and with initial activation of the SN in preparation for task performance. In ADHD, a disbalance of tonic signaling may result in abnormal activation of the DMN, which interferes with consolidation of task-positive networks, and produces overactivation of the VAN and attenuation of the SN (see **Figure 1**).

We will bring together different lines of evidence, from pharmacology, brain imaging and electrophysiology to provide a coherent account of the processing of irrelevant stimuli in ADHD and other conditions. The line of thought of this article starts with an account of the dopaminergic (catecholaminergic) hypothesis of ADHD and how the phasic–tonic signaling dynamics may underlie distinct behavioral states such as focusing, distractibility and impulsivity. Secondly, we characterize the DMN and the task-positive networks: the DAN, the VAN and the SN mentioned above, providing an account of behavioral states that complies with that outlined for catecholaminergic signaling. Third, we review evidence linking deficits in the task-positive network and the DMN in ADHD and other conditions in order to verify an impaired network dynamics in these disorders. Fourth, we review evidence from genetic and pharmacological modulation of the DMN that suggests a link between the catecholaminergic systems and the task-positive/DMN network dynamics. Fifth, an outline of electrophysiological findings that are consistent with the imaging-based evidences for the abovementioned networks will be presented. This is important as it provides a confirmation of the hemodynamic imaging results with a direct electrical measure of the neural activity. Furthermore, we present evidence from event-related potentials that is consistent with an abnormal phasic–tonic signaling and a dysfunction of the VAN–SN, respectively. Finally, we provide a brief discussion in which we synthesize the proposals of this article and point to possible lines of research and therapeutic actions. We hope that this account may contribute to a better clinical understanding and to the search of new therapeutic approaches to this and related conditions.

## A DYNAMIC DOPAMINERGIC HYPOTHESIS OF ADHD

This section provides an overview of the evidence linking dopamine and catecholamines with ADHD, and proposes a relation between phasic–tonic neurotransmitter dynamics and ADHD symptomatology.

### PHARMACOLOGICAL TREATMENT AND GENETICS OF ADHD SUPPORT THE DOPAMINERGIC HYPOTHESIS

In both children and adults, ADHD is treated in the first instance with stimulant medications (which facilitate dopaminergic and noradrenergic neurotransmission), with benefits in 70–90% of patients. Atomoxetine, a non-stimulant drug that acts primarily on the noradrenergic system is used as a second option (Aboitiz et al., 2010). In addition, there is evidence of a weak but consistent link between genetic polymorphisms associated to the catecholaminergic system and ADHD. Specifically, the seven-repeat allele in the D4 receptor (DRD4-7R), the 10-repeat variant in the dopamine transporter (DAT1) gene, and other polymorphisms in the D2 and D5 dopaminergic receptors, and in the gene coding for catechol-*O*-methyl-transferase have been associated with ADHD (Carrasco et al., 2006; Faraone and Khan, 2006; Swanson et al., 2007). This evidence prompted the widely accepted hypothesis that in this condition there is an underlying deficit in catecholaminergic, especially dopaminergic, neurotransmission (Clark et al., 1987), although there are dissenting opinions (Singh, 2008; Gonon, 2009). Furthermore, the dopaminergic system modulates neural systems that are associated with ADHD symptomatology, primarily fronto-striatal circuits involved in predicting events and selecting motor commands (Swanson et al., 2007; Liston et al., 2011; Sato et al., 2012). Secondly, dopamine also affects the fronto-amygdalar circuit processing the emotional contents of events and actions (Swanson et al., 2007). In fact, a recent proposal separates the “cool symptoms” (those mainly cognitive) from the “hot” symptoms (those emotionally related) as two distinct components contributing to the diversity of ADHD symptoms (Castellanos et al., 2006).

Supporting the dopaminergic hypothesis, stimulant medication reinforces dopaminergic signaling and partially restores the executive deficits in ADHD. There is ample evidence for the short-term efficacy and tolerability of stimulants and related compounds like atomoxetin in this condition (Rappley, 2005; Findling, 2008; Stein, 2008; Aboitiz et al., 2010). Furthermore, many imaging studies have demonstrated a significant normalization in the activity of fronto-striatal regions and networks that are dysfunctional in ADHD (Kelly et al., 2009; for reviews, see Swanson et al., 2007; Liston et al., 2011; Spencer et al., 2013). Nonetheless, other evidences point to other neurotransmitter systems besides catecholamines, such as serotonin and actetylcholine in the symptomatology of this condition (Swanson et al., 2007; Aboitiz and Castellanos, 2011). The recent finding of a close link between a polymorphism of the latrophylin 3 gene and ADHD (Arcos-Burgos et al., 2010), and the finding that a knock-out of this gene produces hyperactive behavior and deficits in the dopaminergic system in the zebrafish (Lange et al., 2012) suggests something in this line.

### TONIC AND PHASIC DOPAMINERGIC SIGNALING HAVE DISTINCT BEHAVIORAL CORRELATES

The symptomatology of ADHD is complex and heterogeneous, and a simple dopaminergic or catecholaminergic insufficiency may not account for this diversity. Some PET studies suggest a lower basal dopaminergic tone in ADHD, based on a higher density of the dopamine transporter DAT1 in non-treated patients, which is normalized after medication (Krause et al., 2000; Spencer et al., 2005). Other studies indicate a decreased density of D2-like receptors in non-treated patients, which apparently correlates with inattention symptoms (Volkow et al., 2007a,b). Nonetheless, it has to be mentioned that other reports have found no differences, or even a decreased density of the DAT1 transporter (van Dyck et al., 2002; Jucaite et al., 2005). Part of these discrepancies may be due to the specific radioligand used in each case, as these compounds usually have cross-reaction with other transporters.

Perhaps more important than an overall dopaminergic deficit, a disbalance of different modes of dopaminergic transmission may relate to ADHD symptomatology (Aboitiz and Castellanos, 2011). A crucial function of catecholamines, and particularly dopamine, relates to the execution of goal-directed behavior, which simply put is the ability to appropriately respond to stimuli that predict future events, thereby orienting behavior to approach (or in some cases avoid) these events. As mentioned above, there are two main types of dopaminergic (and noradrenergic) neurotransmission (Grace et al., 2007). Phasic transmission consists of strong but short-lasting trains of stimulus-related dopamine release; this has been considered to be mediated mainly by D1-like dopaminergic receptors in the case of dopamine. On the other hand, tonic liberation refers to the maintenance of basal levels of extrasynaptic neurotransmitter, and is mediated by D2-like receptors (dopamine). As said, phasic activity is mediated by salient or motivating stimuli, while tonic activity defines a basal level of dopaminergic activity. An appropriate, alternating balance between phasic and tonic activity allows the initial focalization of behavior according to relevant stimuli (phasic transmission), while different levels of basal tonic activity may permit to maintain the focus over time as well as updating information in a changing context (Seamans and Yang, 2004). It is likely that norepinephrine, another catecholamine, works in a very similar manner (Aston-Jones and Cohen, 2005).

### A GRADUAL INCREASE IN TONIC ACTIVITY IS ASSOCIATED TO PRE TASK EXPECTANCY

Tonic activity gradually increases with the expectancy of an uncertain event, associated with alertness and preparation for an outcome (Quartz, 2009). There is an interesting interaction between phasic and tonic signaling during tasks where the reinforcer is presented with some uncertainty (for example, the predicting stimulus is associated to the expected outcome – reward – in only 50% of the cases instead of 100%). In this case, phasic dopamine liberation, that is normally associated to the predicting stimulus, triggers a steady increase in tonic dopaminergic levels in the interval between stimulus and outcome, which acquires a maximal slope with maximal uncertainty (Schultz, 2007; Quartz, 2009). In other words, phasic liberation increases linearly with the probability of reinforcement, while tonic liberation displays a bell-shaped curve with a maximum at probability 0.5 of reinforcement appearance. Tonic liberation in the interval between stimulus and reward has been associated to uncertainty-related expectancy (Schultz, 2007; see also Fallon et al., 2013). In other words, stimulus-related phasic activity sets the behavioral and executive systems on, while the maintenance of alertness and behavioral preparation depends on the gradual increase of tonic activity that suppresses further bursts of phasic liberation. Nonetheless, while a moderate increase of tonic activity may facilitate to keep active the representation of the objective and increase performance, too high levels result in anxiety and unrestlesness (Quartz, 2009; Aboitiz, 2009a,b).

### ADHD SYMPTOMS RELATE TO CATECHOLAMINERGIC DYNAMICS

In this context, we have previously proposed that in ADHD there is a disbalance between both signaling mechanisms, sometimes generating states of high impulsivity due to emphasis on phasic liberation (Grace et al., 2007). On the other hand, a dysregulation of tonic dopamine levels might generate distractiveness or anxiety when too low or too high, respectively (Aboitiz, 2009a,b; Aboitiz and Castellanos, 2011). A key point here is that an incapacity to maintain appropriate tonic activity in a determined setting may result in a low threshold of phasic activity and an instability of large-scale brain networks controlling behavior. For example, the ADHD-risk DRD4-7R polymorphism has been proposed to be a hypofunctional form that blunts tonic dopaminergic signaling in the prefrontal cortex. This interferes with the mechanisms involved in preparation and expectancy to future events (Wang et al., 2004). This proposal is consistent with a decreased arousal of ADHD children during task performance (Sergeant, 2000; Ortega et al., 2013). Other authors have proposed that high levels of tonic dopamine maintained chronically, lead to a downregulation of D2-like autoreceptors that control phasic activity. This increases the phasic response to novel stimuli and favors impulsivity (Zald et al., 2008).

Importantly, we have to recall that recent evidence points to noradrenergic signaling (another catecholamine) in ADHD symptomatology. Norepinephrine modulates locomotor activity, arousal, attention, shifting of set, and cooperates with dopamine in working memory tasks (Arnsten, 2007). Norepinephrine also displays a phasic/tonic dynamics (Clayton et al., 2004). Furthermore, tonic locus ceruleus activity (the main productor of brain noradrenaline) has been related with distractibility and to ADHD (Aston-Jones and Cohen, 2005).

### SUMMARY: ADHD SYMPTOMS MAY BE BETTER ACCOUNTED BY CONSIDERING CATECHOLAMINERGIC DYNAMICS

Evidence from pharmacotherapy, genetics and brain imaging has suggested a deficit in basal catecholaminergic signaling in ADHD. However, symptomatology of this condition is diverse and catecholaminergic signaling is also complex. The dysregulation of basal, tonic catecholaminergic levels may account for symptoms of distractibility if too low, and hyperactivity and anxiety if too high. On the other hand, abnormal phasic signaling may correlate with impulsivity and distractiveness to irrelevant stimuli.

## NETWORK MODELS OF ATTENTIONAL REGULATION

In this section, we review the evidence for a DMN whose activity opposes with task-positive networks that participate in attentional engagement. We also discuss distinct components of the task-positive networks, notably the VAN and the SN.

### THE DEFAULT MODE NETWORK OPPOSES TO THE TASK-POSITIVE NETWORKS

**FIGURE 2:**
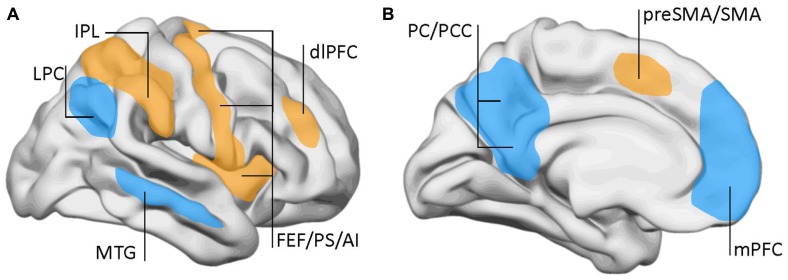
**Lateral (A) and mid-sagittal (B) views of the brain showing the areas involved in the default-mode network (DMN) and the task-positive network.** DMN (blue): LPC, lateral parietal cortex; mPFC, middle prefrontal cortex; MTG, mid-temporal gyrus; PC, precuneus; PCC, posterior cingulate cortex. Task-positive areas (yellow/orange): AI, anterior insula; dlPFC, dorsolateral prefrontal cortex; FEF, frontal eye fields; IPL, inferior parietal lobe (anterior aspect); preSMA, pre somatomotor area; PS, precentral sulcus; SMA, somatomotor area. Data from Raichle et al. (2001) and Fox et al. (2005).

Using fMRI, the DMN was initially described by Marcus Raichle and collaborators as a set of brain regions that were most active during restfulness and became suppressed when the subject engaged in a cognitive task (Raichle et al., 2001; Raichle, 2010). The DMN encompasses the medial prefrontal cortex, the posterior cingulate, or precuneus and the posterior parietal cortex among other regions (**Figure 2**). This was distinguished from the task-positive network, which shows increased activation during cognitive or executive engagement. The task-positive network includes brain regions involved in motor planning and executive functions like the dorsolateral prefrontal cortex, the frontal eye fields, premotor, and inferior parietal areas.

The DMN was eventually found to be related to processes like introspection, autoreferential activity, mentalization, and some aspects of social behavior (Qin and Northoff, 2011). Furthermore, a within-network synchronic oscillatory activity of the DMN was evidenced, which noticeably was strictly antisynchronic with the oscillatory activity of the task-positive regions. That is, when one network goes up the other goes down and vice versa (Fox et al., 2005; Kelly et al., 2008). Moreover, it was found that during a continuous performance task the variability of response times was correlated with the oscillatory activity of the DMN. Longer reaction times corresponded to instances where the stimulus was presented during a state of high activity of the DMN and vice versa, shorter reaction times were associated with decreased activity of the DMN (Weissman et al., 2006; Fox et al., 2007).

### THE DORSAL ATTENTIONAL NETWORK AND THE VENTRAL ATTENTIONAL NETWORK

**FIGURE 3:**
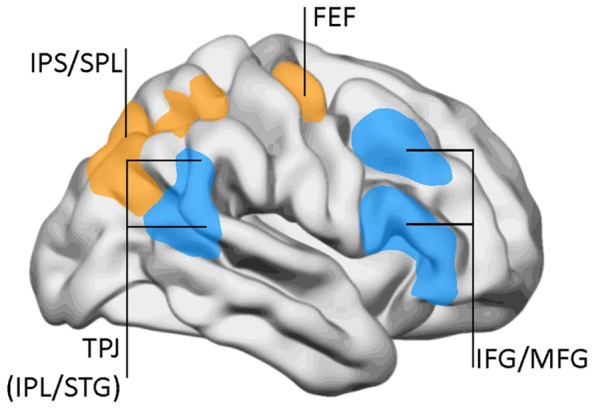
**The dorsal attentional network (DAN) and the ventral attention network (VAN).** DAN (yellow/orange): FEF, frontal eye fields; IPS, inferior parietal sulcus; SPL, superior parietal lobe. VAN (blue): IFG, inferior frontal gyrus; IPL, inferior parietal lobe (posterior aspect); MFG, middle frontal gyrus; TPJ, temporo-parietal junction; STG, superior temporal gyrus. Data from Corbetta and Shulman (2002), with permission.

The task-positive network is not a unitary system, but is divided in different subnetworks. By the same time as the DMN was identified, Corbetta and collaborators proposed a two-component model for top-down and bottom-up attentional control (Corbetta and Shulman, 2002; Corbetta et al., 2008). Top-down influences involve a DAN comprising the superior parietal lobe, intraparietal sulcus, and frontal eye fields (**Figure 3**). This network has a significant overlap with the task-positive networks described by Raichle and collaborators, especially in the dorsal parietal and frontal regions. On the other hand, the VAN conveys bottom-up influences and mediates changes in attentional focus, and two-way transitions between the default and executive networks. The VAN is strongly lateralized to the right, and comprises the temporo-parietal junction (supramarginal and superior temporal gyri) and the medial and inferior frontal gyri. As with the default network, the metabolic activity of the VAN decreases during focused states, but becomes transiently activated during attentional reorientation.

The role of the VAN during task performance, attentional reorienting, and goal-directed behavior remains enigmatic because its temporal dynamics and link to behavior are still poorly understood. One hypothesis is that the VAN works as a circuit-breaker that interrupts ongoing cognitive activity, by modulating dorsal network selection when an unexpected behaviorally relevant event is at hand (Corbetta et al., 2008). However, it is not clear where the reorienting signal initiates, and whether the interaction between the two systems occurs directly or through connections with sensory areas (Ossandón et al., 2011). Although reorienting to behaviorally relevant events is critical for survival, reorienting to irrelevant stimuli may interfere with task performance. Hence, during demanding cognitive engagement, it may be advantageous to impose an attentional filter that restricts VAN activation, protecting the ongoing focus of attention from distractors (Corbetta et al., 2002; Shulman et al., 2002; Anticevic et al., 2010). We will hold to this model as we consider it fits some ADHD symptoms, particularly the distractibility to irrelevant stimuli.

### THE SALIENCE NETWORK

An additional proposal has pointed to a “SN” involving dorsal anterior cingulate regions and orbital frontoinsular cortex that dissociates from the DAN, although both together suppress the DMN (Seeley et al., 2007). The SN activates with emotionally salient and behaviorally relevant stimuli, and has been related with anxiety, expectation and the fight or flight reaction (Seeley et al., 2007; Bressler and Menon, 2010; Chen et al., 2013). Activation of this network may thus be associated with tonic increases of catecholamines during expectation for a task (see Section The Dorsal Attentional Network and the Ventral Attentional Network).

### OTHER MODELS FOR ATTENTIONAL CONTROL

The above described networks are partly consistent with an early model for attentional regulation, described by Posner and collaborators (Posner and Petersen, 1990; Petersen and Posner, 2012). Based on performance on the attentional network test (ANT), these authors described three different networks involved in attentional processing: (i) the alerting network related to brainstem arousal modulators (particularly norepinephrine) that act diffusely in the brain; (ii) the orienting network that generates attentional displacements and includes the frontal eye fields and the intraparietal sulcus, which is modulated by acetylcholine (this network overlaps with both the DAN and the VAN); and (iii) the executive network, involving midline components (regulated by dopamine). Subsequently, the executive network became subdivided into a frontoparietal control system (allegedly distinct from the orienting network, but nonetheless strongly overlapping with it), which participates in moment-to moment control during a task; and a cingulo-opercular network (that overlaps with the SN) related to task set maintenance (Petersen and Posner, 2012). The orienting network is overactivated at early ages but as the child matures it becomes progressively modulated by the executive network, gradually developing the capacities for effortful control and social development (Petersen and Posner, 2012). In our view, ADHD symptomatology fits this scheme, with an overactive orienting network and a poorly developed executive network compared to the general population at the same age.

### SUMMARY: FOUR MAIN ATTENTIONAL SYSTEMS, DAN, VAN, SN, AND DMN

Although there is no strict consensus on the separation of different attentional systems, for the purpose of this article we will propose four main networks regulating attention. By combining the different models of attentional networks, we suggest the following classification (**Figure 1**). The task-positive network involves the DAN that participates in working memory and sustained attention; the second is the VAN, involved in attentional shifts, and the third is the cingulo-opercular, SN involved in emotional responses and expectation. The default network (DMN) involved in introspection, acts opposite to the others. While the DAN, the SN and the DMN imply sustained activations and may relate to tonic levels of neurotransmitters like catecholamines, the VAN is activated transiently in response to internal or external stimuli and may relate with phasic neurotransmission. Phasic responses to salient stimuli may also activate the SN, associated with a gradual increase of tonic activity during expectation.

## THE DMN IN ADHD AND OTHER CONDITIONS

This section discusses evidence that the balance between the task-positive networks and the DMN is altered in ADHD and other neuropsychiatric disorders, which may be interpreted to be consequence of a dysregulation of tonic dopaminergic signaling. Evidence for involvement of the VAN and the SN in ADHD is still pending for future research.

### IN ADHD THERE IS A BREAKDOWN OF ANTISYNCHRONY BETWEEN THE DMN AND THE TASK-POSITIVE NETWORKS

Partly based on the above evidence, Sonuga-Barke and Castellanos (2007) proposed that in ADHD and other neuropsychiatric conditions the DMN would maintain an activated state, interfering with the maintenance of task-positive networks required for efficient cognitive and executive processing. This proposal is supported by the high variability in reaction times observed in ADHD in continuous performing tasks (Castellanos et al., 2005). Many studies suggest a decrease in antisynchrony between the task-positive and task-negative networks (Fassbender et al., 2009; Sun et al., 2012; Hoekzema et al., 2013), while others report a within-network decrease in functional connectivity of the default network in ADHD patients (Carmona et al., 2005; Rubia et al., 2005; Cao et al., 2006; Tian et al., 2006; Makris et al., 2007; Uddin et al., 2008; Broyd et al., 2009). One possibility, consistent with both views, is that a lack of cohesion within the DMN results in a disorganized dynamics that cannot be effectively suppressed when the subject engages in a cognitive task. Furthermore, a disorganized DMN may be associated to an increased excitability of the VAN, yielding distractibility to irrelevant stimuli.

Supporting the above interpretation, a few recent studies have specifically addressed the antagonistic interactions between the task-positive and DMN networks in ADHD. In a report on functional connectivity during rest, Sun et al. (2012) evidenced a decreased anti-correlation between the dorsal anterior cingulate cortex and the DMN in boys with ADHD respect to typically developing subjects. In addition, Hoekzema et al. (2013) observed a stronger coherence between activities in the dorsolateral prefrontal cortex and the DMN in ADHD than in controls, which was correlated with impaired performance in tasks of selective attention. Likewise, in ADHD there was a reduced anticorrelation between the dorsolateral prefrontal cortex and the DMN. These authors interpret these findings as due to an intromission of the DMN into the task-positive network together with a weaker inhibition of the dorsolateral prefrontal cortex during rest. This results in the breakdown of the anticorrelation between the task positive and the DMN networks and suggest that anti-correlations are key to understanding deficits in ADHD.

### DECREASED FUNCTIONAL CONNECTIVITY OF THE DMN IN ADHD

In addition, there are several reports indicating an abnormal connectivity of the DMN in ADHD subjects. For example, in resting-state adults with ADHD, Castellanos et al. (2008) observed a decrease in functional connectivity between the anterior cingulate cortex and the precuneus/posterior cingulate cortex and between the precuneus and both the ventromedial prefrontal cortex and posterior cingulate cortex. More recently, Fair et al. (2010) detected a reduced within-network connectivity in ADHD at rest compared to controls, and Sato et al. (2012) assessed an abnormality index for functional connectivity in fMRI-based resting-state low-frequency oscillations (0.05–0.2 Hz). The dorsal anterior cingulate cortex and the posterior cingulate cortex displayed a higher abnormality index for adult ADHD than for typical developing subjects, but this value was similar between adult ADHD patients and typical developing young subjects. This points to and immaturity of the DMN and task-positive networks and their interactions in ADHD. Using resting-state fMRI, Cao et al. (2009) detected a positive functional connectivity between the putamen and sensorimotor, prefrontal, insula and other cortical regions, and a negative functional connectivity between the putamen and parietal, occipital cortices and other brain regions. In this study, ADHD children had a reduced functional connectivity between cortical regions and the putamen, but not between the putamen and globus pallidus. Interestingly, Tomasi and Volkow (2012) reported in ADHD a lower than normal overall connectivity (long and short-range) in both the dorsal attention network and the DMN, but higher short-range connectivity in the ventral striatum and orbitofrontal cortex. These findings imply an emphasis in reward-related connectivity at the expense of task-positive and resting state networks in ADHD.

Furthermore, functional and structural alterations in the posterior cingulate cortex, precuneus and medial prefrontal cortex of persistent ADHD adults suggest that the DMN plays a role in the maintenance of this condition throughout life, in which inattentive symptoms predominate (De La Fuente et al., 2013). On the other hand, the absence of striatal structural alterations might reflect the maturation of these structures associated to the vanishing of hyperactive symptomatology in adulthood (De La Fuente et al., 2013).

### THE DMN IS ALTERED IN OTHER NEUROPSYCHIATRIC CONDITIONS

Beside ADHD, deficits in the DMN have been reported in several conditions, including dementia, schizophrenia, epilepsy, anxiety and depression, and autism (Broyd et al., 2009). There are reports of DMN dysfunction in Alzheimer’s and Parkinson’s diseases, although in the latter there are some conflicting data (Rektorova, 2013). However, Delaveau et al. (2010) found that Parkinson’s patients fail to deactivate posterior midline or lateral components of the DMN during a facial emotion recognition task*.* In schizophrenia and bipolar disorder, there are also reports indicating a deficit in the DMN (Ongür et al., 2010; Orliac et al., 2013). A lower than normal mean network homogeneity (a measure of the correlation of a given voxel with other voxels in a given network) was evidenced in the resting state of first-episode schizophrenic subjects, particularly in the medial prefrontal cortex and middle temporal gyrus (Guo et al., 2013); however, in the posterior cingulate cortex and cerebellum this measure reached higher than normal levels (Guo et al., 2013). In autistic patients, Assaf et al. (2010) reported a decreased resting state functional connectivity between precuneus and medial prefrontal and anterior cingulate cortices, while Lynch et al. (2013) observed hypoconnectivity between precuneus and posterior cortical regions and basal ganglia but hyperconnectivity between posterior cingulate/retrosplenial cortices and the anterolateral temporal cortex. Interestingly, Christakou et al. (2012) observed a shared lack of deactivation in the precuneus during an sustained attention task in both ADHD and autistic patients, but more underactivation of the left dorsolateral prefrontal cortex in ADHD than in autistic patients, which was associated with impaired sustained performance in the former. On the other hand, autistic children showed a specific increase in cerebellar activation during the task. More recently, Washington et al. (2013) found that children with autistic spectrum disorder have reduced connectivity between DMN nodes but increased connectivity within nodes. While between-node connectivity gradually increases with age in normal children, such connectivity remains impaired in adolescent autists. However, other findings indicate greater functional connectivity between the right lateral parietal cortex and the anterior medial prefrontal cortex, both components of the DMN (Redcay et al., 2013). In general, it seems evident that defects in the DMN and its relation with the task-positive networks are not exclusive of ADHD, but can nevertheless explain much of the symptomatology in this condition.

### SUMMARY: ALTERATIONS OF DMN DYNAMICS OCCUR IN ADHD AND OTHER CONDITIONS

There is a lot of recent evidence indicating a dysregulation of the DMN in ADHD and other neuropsychiatric disorders. Nonetheless, little study has been made on the activity of the VAN or the SN in these conditions. Our proposal consists of highlighting these networks as possible substrates for ADHD symptomatology.

## GENETIC AND PHARMACOLOGICAL MODULATION OF THE DMN

Here we discuss genetic and pharmacological influences in DMN activity are consistent with a neurochemical modulation of the DMN and the alternating dynamics with task-positive networks.

### POLYMORPHISMS OF THE DOPAMINERGIC SYSTEM HAVE DIFFERENTIAL EFFECTS ON THE DMN

Although imaging genetics is still in its infancy (Durston, 2010), there have been a few studies that address the modulatory effects of ADHD-risk genetic polymorphisms on DMN activity. For example, in a PET visual attention task, availability of the dopamine transporter DAT in caudate and putamen has a negative correlation with the deactivation of the posterior components of the DMN and correlates positively with deactivation in the ventral anterior cingulate gyrus (Tomasi et al., 2009). In addition, a study in adults found an association between a dopamine transporter polymorphism, the default network, and ADHD symptoms (Brown et al., 2011). Interestingly, our group has recently observed that the evolution of reaction times along a continuous performance task is dependent on the risk polymorphisms of the dopamine transporter gene (Henríquez-Henríquez et al., 2012). In an fMRI/SPECT study, two genetic polymorphisms of the D2 dopamine receptor (GG and GT) were found to modulate the connectivity of the DMN and striatal connectivity. Subjects homozygous for the G allele displayed greater prefrontal cortex efficiency during cognitive processing than subjects carrying one copy of the T allele (Sambataro et al., 2013). Analysis of resting state activity revealed that homozygotes had higher connectivity in the medial prefrontal cortex, in correlation with increased striatal availability of the presynaptic dopamine transporter; however, their posterior cingulate cortex had reduced connectivity in relation to heterozygotes (Sambataro et al., 2013). Lee et al. (2012) assessed dopamine receptor DRD4 polymorphisms and the DMN in normal subjects. These authors found that subjects bearing long, ADHD-risk DRD4 alleles (>4R) had decreased resting state EEG connectivity strength, bilaterally in frontal–parietal and in right temporo-frontal connections (Lee et al., 2012). Overall, these findings support the notion that genetic ADHD-risk factors are associated with a decrease in connectivity and lack of deactivation of the DMN.

### GABA AND STIMULANTS CONTRIBUTE TO ATTENTIONAL NETWORK TRANSITIONS

Neurochemical modulation of the DMN and task-positive networks is to be expected, but very little information has yet been collected in this topic. Interestingly, using MR spectroscopy, Hu et al. (2013) recently observed that in normal subjects, high GABA concentration in the posterior cingulate–precuneus region correlated with increasing task-induced deactivation of these regions; conversely, high glutamate concentrations were related with reduced deactivation. In addition, stimulants have been found to enhance task-related DMN deactivation and to partially restore the DMN dysfunctions in ADHD. Minzenberg et al. (2011) measured the modulating effects of modafinil, a norepinephrine/dopamine transporter inhibitor, in the deactivation of the DMN during an event-related fMRI visual sensorimotor task. Together with increasing behavioral performance, there were several DMN components exhibiting an increased deactivation compared to placebo, like the ventromedial prefrontal cortex, the posterior cingulate and retrosplenial cortices, and the left inferior parietal lobe. Furthermore, the effects of modafinil in the ventromedial prefrontal cortex were specifically associated with a decrease in reaction times during the task. More recent reports indicate that the dopaminergic agonist levodopa increases connectivity between the midbrain and the DMN, between the caudate and frontoparietal networks, and between the ventral striatum and a fronto-insular network (Cole et al., 2013a,b). These effects perhaps contribute to stabilize the dynamics of the DMN and the task positive network.

### STIMULANTS IMPROVE DMN ACTIVITY IN ADHD

Liddle et al. (2011) assessed both motivation and stimulant effects in the DMN of ADHD children and controls during a go–nogo task. Lowly motivated and off-medicated ADHD children displayed a significant DMN deattenuation during the task compared to controls, but there were no differences in the motivated condition. In fact, the effects of motivation were higher in ADHD subjects than in controls. However, under stimulant medication, there was no motivational modulation of DMN attenuation, displaying a similar pattern as in typically developing children. Furthermore, Cubillo et al. (2013) assessed the normalization effects of methylphenidate (MPH, a dopaminergic enhancer) and atomoxetine (ATX, a noradrenergic enhancer) in ADHD boys during an *n*-back working memory task. Both drugs increased fronto-striatal activation and downplayed the DMN. Furthermore, in medication-free ADHD subjects, there was a bilateral inactivation pattern in the dorsolateral prefrontal cortex at high working memory loads. ATX increased the activation of the right DLPFC relative to MPH, while MPH upregulated the left inferior frontal cortex only in the two-back condition. Finally, using MEG, Franzen et al. (2013) evaluated mid- and low-frequency phase coherence among DMN regions in ADHD adults before and after stimulant therapy, compared with age-matched controls. In unmedicated subjects, there was a reduced connectivity between posterior cingulate/precuneus and right inferior parietal cortices, and between the medial prefrontal cortex and the left inferior parietal region and posterior cingulate cortex. This group also displayed stronger phase locking between the right and left inferior parietal cortices in relation to controls. Stimulant administration resulted in an increase of connectivity between the middle prefrontal cortex and the posterior cingulate cortex, and a decrease in connectivity between the left and right inferior parietal cortices.

### SUMMARY: GENETIC AND PHARMACOLOGICAL EVIDENCE FITS DMN ACTIVITY AND ADHD SYMPTOMS

Although very recent, genetic and pharmacological evidence is accumulating that supports a modulatory role of catecholamines and other neurotransmitters in the activity of the DMN and the task-positive networks. This implies that neurochemical signaling may underlie the dynamics of these large-scale cortical networks and be related to ADHD symptomatology.

## ELECTROPHYSIOLOGICAL STUDIES OF THE DMN

In this section we review evidence from frequency analyses in the EEG that provides a direct measure of the DMN. Furthermore, evidence from event-related potentials is consistent with DMN irruption in the attentional focus by abnormal excitability of the VAN, and with a suppressed SN that impedes proper preparation for the task.

### ANALYSES OF EEG OSCILLATIONS HAVE IDENTIFIED A CORRELATE OF THE DMN

Finding a neurophysiological expression of the DMN is crucial in order to confirm and characterize a neural, not hemodynamical source of this activity. In this line, Miller et al. (2009) evidenced a correlation between high-frequency activity (76–200 Hz) in default regions measured by the electrocorticogram, and the default network oscillations observed in fMRI. Similarly, using intracortical recordings, Ossandón et al. (2011) observed a suppression of high-frequency gamma activity (60–140 Hz) in regions associated to the DMN, when subjects engaged in a visual search task. Another line of evidence has come from the analysis of very low frequency electrophysiological oscillations (VLF, 0.02–0.2 Hz; Vanhatalo et al., 2004), that are similar to baseline slow hemodynamic oscillations (0.01–0.08 Hz) displaying decreased amplitude in ADHD (Zang et al., 2007). Several authors have characterized the EEG activity related to the default network on the basis of topographic distributions of such low-frequency oscillations, which have been found to activate–deactivate between rest and task conditions (Laufs et al., 2003; Mantini et al., 2007; Chen et al., 2008; Helps et al., 2008, 2009). In ADHD subjects, there is a reduced power in VLF oscillations compared to controls, as well as a weaker deactivation in rest-to-task transitions (Helps et al., 2010). Furthermore, in low-rating ADHD subjects this deactivation is stronger than in high-rating ADHD subjects, in the medial prefrontal cortex and in temporal regions (Broyd et al., 2011). Nonetheless, neurophysiological alterations in ADHD seem not to be restricted to VLFs but may relate to an overall deficit in broadband activity (Wilson et al., 2013). These authors reported broadband alterations and an abnormal cross-frequency gamma coupling between the medial prefrontal cortex and the posterior cingulate cortex in ADHD adults (Wilson et al., 2013), which were restored after stimulant medication.

### EXPERIMENTS USING EVENT-RELATED POTENTIALS ARE CONSISTENT WITH AN OVERACTIVE VAN AND AN IMMATURE SN IN ADHD

**FIGURE 4:**
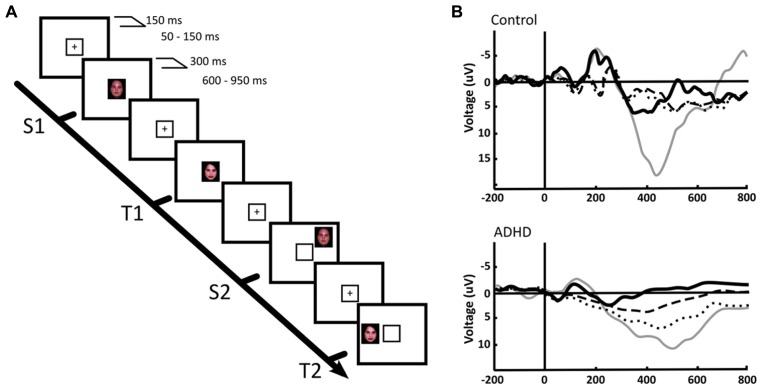
**Central and peripheral attention in ADHD and control children.** **(A) **We used a face recognition, spatial-shifted double oddball task in which a frequent, standard stimulus (S1) was presented within a central frame, alternating with an infrequent, target stimulus (T1). However, in some trials the frequent or the standard stimuli appeared outside the attentional frame (S2, T2, respectively). **(B)** P300 ERPs elicited by the distinct stimuli in a central ROI. Thick continuous line: S1, standard stimulus on attentional focus. Thin continuous line: T1, target stimulus on attentional focus. Dotted line: T2, target stimulus in the periphery. Segmented line: S2, standard stimulus in the periphery. The results indicate that in the focused condition, only the target stimulus (T1) elicits a P300, both in ADHD and controls. However, for peripheral stimuli, a P300 deflection is observed only in ADHD. For further details, see López et al. (2006) with permission.

Beside frequency analyses, event-related potential (ERP) studies have been performed that in our view support the concept of an alteration in the balance between the DMN and the task positive networks, and suggest an overactivation of the VAN in ADHD. In normal subjects, the P300 ERP, which associates to the activation of working memory networks during the execution of a task, has a smaller amplitude in trials preceding erroneous responses and in those where subjects reported to have been mind wandering, in both children and adults (Smallwood et al., 2008). In addition, ADHD (as well as other conditions) is characterized by a significant reduction of the P300 evoked potential amplitude, which restores to nearly normal levels after medication (López et al., 2004). In a first study to assess the response to peripheral, unattended stimuli in ADHD children, we designed two tasks: one in the spatial domain and the other in the time domain. In the first one, we presented an oddball visual recognition task, with a frequent standard stimulus (a face) alternating with an infrequent target stimulus (another face) that had to be recognized. These stimuli (valid stimuli) were presented within a yellow frame at the center of the screen. However, in some trials the same stimuli were presented not in the center but outside the yellow square, in the periphery of the screen (see **Figures 4A,B**). The explicit instruction was to count the number of times that the target stimulus appeared within the central yellow frame (the subject did not have to count standard stimuli), and not to pay attention to the peripheral stimuli (López et al., 2006). In a classical central oddball task, only the target stimulus (the stimulus to be recognized) elicits a P300 potential. However, both the target and the standard stimuli are able to generate the early sensory potentials P100/N100 (although target stimuli produce larger early potentials than standards, especially N100). In our experiment, this response was observed in both ADHD and controls for the central task, only the P300 having smaller amplitude in ADHD subjects. In both ADHD and controls, the peripheral stimuli that we included in our task, that had to be ignored, showed a significant reduction of the early potentials N100/P100 compared to the central stimuli, indicating that the sensory attentional filter was intact in both groups. However, the late potential P300 behaved highly differently in controls and subjects: while peripheral stimuli were unable to elicit a P300 in controls, ADHD subjects evidenced a significant P300 deflection in this condition (**Figure 4B**). This indicates that, despite there being an early sensory suppression of the distracting stimuli, the latter were able to deviate the attentional focus of ADHD subjects, possibly relying on a low threshold for VAN activation and a weakly suppressed DMN. This evidence is consistent with the fMRI finding that in normal subjects, reductions in attention associated to longer response times, are also related to increased activation triggered by stimuli that are irrelevant for the task (Weissman et al., 2009).

**FIGURE 5:**
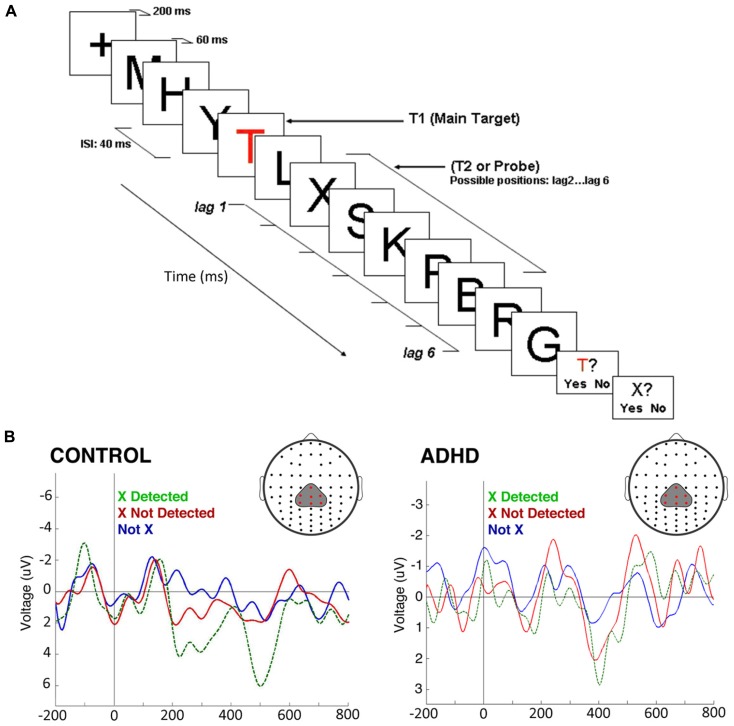
**The attentional blink in ADHD and controls.** **(A)** The task consists of a rapid serial visual presentation paradigm in which there is a salient, colored letter (T) followed by several letters, among them an X which may or may not be present, at different distances from the “T.” Detection of the second letter (“X”) is minimal when it is presented, as shown in the figure, some 80 ms after the “T.” **(B)** ERPs produced by the second letter (“X”) during the attentional blink. ERPs show a morphology that is characteristic of steady-state evoked potentials. After subtracting the effect of the P300 elicited by the first letter (“T”), a P300 was present in control subjects only when they detected the second letter (“X”; green dotted line). If this was present but unnoticed, there was no P300 elicited (red solid line). In ADHD, P300 were of significantly smaller magnitudes [*F*_(1,22)_ = 17.64, *p* < 0.01], as expected, but were present both when the second letter (“X”) was detected, and when it passed unnoticed (note the magnification of voltage scale in the ADHD group, in order to visualize better the differences between conditions). When a letter different from “X” was present, there was no P300 in either group (blue line). The P300 observed displays a longer latency than usual due to the experimental design (a previous relevant stimulus and a steady sate visual ERP paradigm, which produce an increased delay in the stimulus-driven response). Between-group comparisons of P300 latency and topography were not statistically significant. For further details, see López et al. (2008) with permission.

In a second experiment we used a rapid serial visual presentation paradigm to assess attentional mechanisms in the time domain (**Figure 5A**). In this task, we took advantage of the attentional blink phenomenon, where the detection of a second target stimulus is impaired if it is presented too close to the first target (López et al., 2008). Here, controls displayed a robust P300 after the second stimulus only in the case it was consciously detected; if it was present but passed unnoticed, the P300 was not elicited. On the other hand, in ADHD subjects, there were smaller amplitude P300s, but these appeared both when the stimulus was consciously detected and when it was not detected. No P300 was observed in either controls or ADHD subjects when the second target was replaced by a different, irrelevant stimulus (**Figure 5B**). An interpretation of these studies is that in ADHD the attentional focus is incapable of sealing properly, being susceptible to intromission of irrelevant stimuli, which again is consistent with the concept of a DMN interference on the task-positive network, or with an overactive VAN due to low threshold for phasic dopaminergic signaling.

**FIGURE 6:**
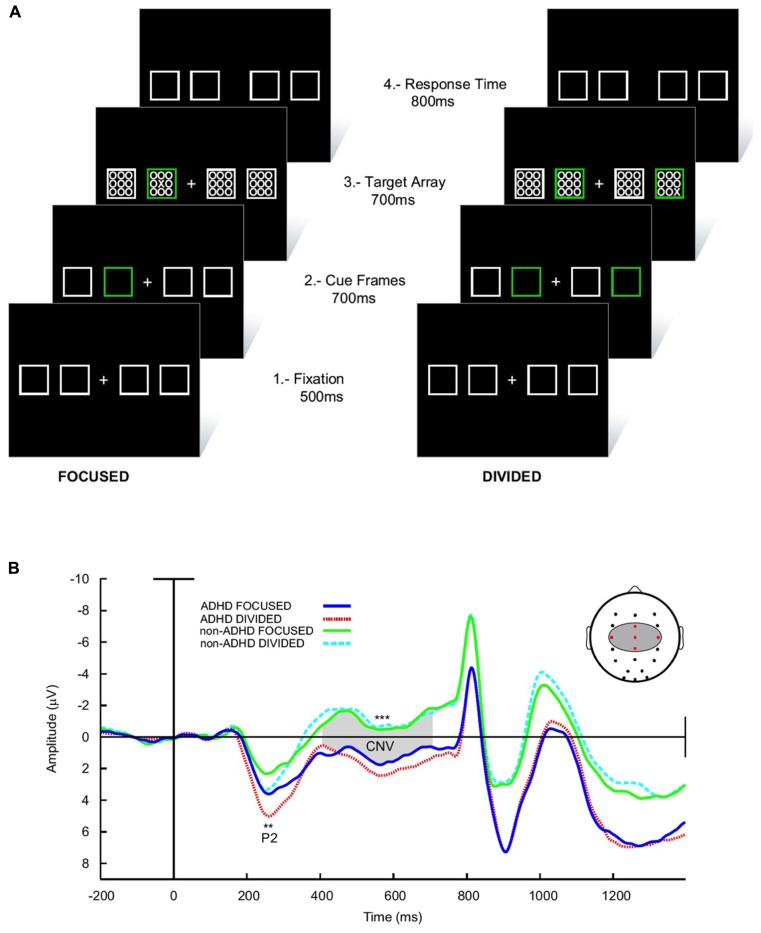
**Preparatory activity during a visual search task in ADHD and controls.** **(A)** The subject had to fixate in front a cross flanked by four squares. Subsequently, one (low attentional load) or two (high attentional load) of these squares lighted in a different color in order to deviate attention. After this, an array of zeroes was presented inside the squares, among which there was also an “X” that had to be detected. **(B)** ERPs elicited during the task. The array of zeroes in the frame was presented at about 800 ms after the presentation of the cue frames. Between 300 and 800 ms, a contingent negative variation (CNV) potential develops, which is indicative of expectancy for the task. Note that in ADHD the CNV is noticeably lower than in controls. No differences in the CNV were seen between the high and low attentional load conditions. For further details, see Ortega et al. (2013) with permission. **p < 0.005; ***p < 0.001.

Finally, in a delayed visual search paradigm, where the task is preceded in 700 ms by a preparatory signal, normal children develop a steady increase in negativity in the EEG (the contingent negative variation, CNV) in the interval between the signal and task onset (**Figures 6A,B**; Ortega et al., 2013). This has been interpreted as a preparation potential. Interestingly, this potential behaves similarly to the above described tonic dopaminergic “ramp” observed in conditioning experiments in animals. In ADHD children however, the CNV is clearly deficient. Furthermore, across subjects the electrophysiological variable that best correlated with task performance was precisely the amplitude of the CNV potential. This evidence points to a deficit in the preparatory mechanisms for a given task in ADHD, which is consistent with the concept of a dysfunction of tonic dopaminergic activity, and notably, with a deficit in the SN described above. In addition, the incapacity to build up an adequate preparedness for the task complies with the proposal that the executive networks are not well stabilized, possibly due to interference from the DMN.

### SUMMARY: EEG CONFIRMS DMN DYSFUNCTION IN ADHD

The fMRI signal is an hemodynamic, indirect measure of neural activity. The neuronal character of the DMN has been recently confirmed, by observing suppression of high-frequency EEG activity in DMN regions during task engagement, and by the detection of VLF oscillations that correlate with behavioral states. In ADHD, there is evidence for a decreased DMN EEG signal in low-frequency oscillatory activity (but also in higher frequencies). In addition, ERP studies have revealed an incapacity to protect the attentional focus from interfering stimuli, possibly associated to abnormal VAN excitability, and a difficulty to build up an executive network in preparation for a task, which complies with the notion of an immature SN.

## CONCLUSION: ABNORMAL CATECHOLAMINERGIC SIGNALING MAY UNDERLIE ALTERATIONS OF THE DMN IN ADHD

Considering the above evidence, it is tempting to propose an association between the phasic/tonic dopamine signaling mechanisms and the task-positive/task-negative network dynamics (Aboitiz and Castellanos, 2011; see **Figure 1**). Increasing levels of tonic dopamine have been related to attentional engaging and goal oriented behavior, while low basal tonic levels may relate to a default activation. In contrast, phasic dopamine is by definition highly transient, and may be more strictly associated to transitions between the resting mode and the task-positive mode (initial activation of the SN) and to attentional shifts during a task (the VAN).

We propose that in ADHD there is a disbalance both in catecholaminergic signaling mechanisms and in the DMN-task positive network interactions, which may be causally related. A dysregulation of basal catecholaminergic activity may result in an incapacity to maintain appropriate tonic levels in specific circumstances, which in turn yields a dysregulation of phasic activity. As a consequence there is a disbalance of the DMN, an increased sensitivity of the VAN and loss of the anticorrelation between DMN and task-positive areas. Behaviorally, this is reflected in an increasing susceptibility to distraction by irrelevant or salient stimuli during a task. Phasic signaling may also trigger a transient increase of tonic activity during expectation and be related to SN activity. However, in ADHD, dysregulation of phasic activity and immaturity of the SN may yield two effects: (i) insufficient buildup of tonic activity, impairing preparation for a task (Ortega et al., 2013), or (ii) an overactivation of the SN yielding impulsivity (Grace et al., 2007; Zald et al., 2008). In our view, a more detailed characterization of the VAN and the SN, and their relations with the DMN in both healthy and ADHD subjects, would significantly contribute to the understanding of attentional dynamics in normal and neuropsychiatric patients.

Finally, even if neurotransmitter dysfunctions may be at the basis of ADHD and other neuropsychiatric conditions, we consider highly valuable alternative therapeutic approaches using cognitive rehabilitation (Rutledge et al., 2012) or brain–computer interfaces (Lim et al., 2012) in which the dynamics of the DMN, task-positive network and VAN can be modulated top-down in order to control dopaminergic abnormal functioning.

## Conflict of Interest Statement

The authors declare that the research was conducted in the absence of any commercial or financial relationships that could be construed as a potential conflict of interest.
